# Farewell letter from Prof. Alexander Tenenbaum, Editor-in-Chief

**DOI:** 10.1186/s12933-021-01424-1

**Published:** 2021-12-13

**Authors:** Alexander Tenenbaum

**Affiliations:** Sheba Medical Center, Tel Hashomer, Ramat Gan, Israel

The close interrelationship between cardiovascular diseases and alterations in glucose metabolism has raised the prospect that atherosclerosis and type 2 diabetes share a common background. Both conditions seem to be based on similar genetic and environmental precursors; this has been termed "common soil" and the American Heart Association explicitly stated in 1999 that "diabetes is a cardiovascular disease".

In this context, in 2001 my friend and colleague Prof. Enrique (Zvi) Fisman and I began to consider the possibility of creating a scientific journal specifically dedicated to cover the intersection between diabetes and cardiovascular disorders. The task did not seem to be easy since it was necessary to establish an Editorial Board, obtain an ISSN (International Standard Serial Number), register the Journal in PubMed and other repositories of medical information, find and appropriate publisher, publicize the existence of the Journal as widely as possible… and we had no idea how to start and accomplish these tasks.

We contacted then BioMed Central (BMC), a United Kingdom-based scientific open access publisher. BMC was then a young and dynamic company, founded in 2000; BMC representatives helped us in each of the initial steps, providing us with guidance and advice. We named the Journal Cardiovascular Diabetology, it was launched on April 2002 and got its first official Journal Impact Factor (JIF) in 2007. BMC is a part of Springer Nature since 2008.

As of January 1, 2022, for personal reasons, I will be compelled to step out of my position as Editor-in-Chief. I have taken this decision with sadness, but at the same time with pride as I look at what has been done during these almost two decades. During this period Cardiovascular Diabetology has raised its JIF in a nearly constant yearly pattern, presenting a 2020 JIF of 9.951, ranking it in the 93.45 JIF percentile in the category “Endocrinology & Metabolism”—being thus the leading open access journal in the field—and in the 90.49 percentile in the category "Cardiac & Cardiovascular Systems” journals. The Journal is offered more articles for publication each year and the number and quality of the published articles grow continuously.

The Journal is currently facing two new challenges. The first is the recruitment of highly qualified Associate Editors, who will help Cardiovascular Diabetology to publish much more articles and in a quick way. The second is the implementation of SNAPP, the new and sophisticated editorial submission system from Springer Nature, that makes articles’ management smoother and more efficient.

None of the above-mentioned achievements would have been possible without the efforts, loyalty and help of countless people. I am aware that the success of our journal is a joint attainment of researchers, authors, readers, Editorial Board members, external reviewers, Journal Development Editors and BMC and Springer Nature scientific and technical teams. I would like to express my gratitude to all of them. It has been an honor and a privilege for me to have been a leading part of this collective initiative.

Thank you.

